# Fabrication of immiscible Cu-V alloy by high-pressure torsion

**DOI:** 10.1007/s44492-025-00002-w

**Published:** 2025-12-01

**Authors:** Serkan Öğüt, Tahereh Zargar, Tayebeh Mousavi, Lubiana Georges, Sumit Ghosh, Atef Hamada, Walaa Abd-Elaziem, Yi Huang, Terence G. Langdon

**Affiliations:** 1 Department of Mechanical Engineering, Faculty of Engineering, Marmara University, Maltepe, 34854 İstanbul Turkey; 2 Department of Engineering, King’s College London, London, WC2R 2LS UK; 3 Polytech Montpellier, University of Montpellier, 34090 Montpellier, France; 4 Materials and Mechanical Engineering, Centre for Advanced Steels Research, University of Oulu, 90014 Oulu, Finland; 5 Kerttu Saalasti Institute, Future Manufacturing Technologies (FMT), University of Oulu, Pajatie 5, 85500 Nivala, Finland; 6 Department of Mechanical Design and Production Engineering, Faculty of Engineering, Zagazig University, P.O. Box 44519, Zagazig, Egypt; 7 Department of Materials Science and Engineering, Northwestern University, Evanston, IL 60208 USA; 8 Department of Design and Engineering, Faculty of Science and Technology, Bournemouth University, Poole, Dorset, BH12 5BB UK; 9 Departments of Aerospace & Mechanical Engineering and Materials Science, University of Southern California, Los Angeles, CA 90089-1453 USA

**Keywords:** Copper, High-pressure torsion, Severe plastic deformation, Ultrafine-grained materials, Vanadium

## Abstract

This study describes the fabrication of immiscible Cu-V alloys through the application of high-pressure torsion (HPT). For this purpose, stacked Cu/V/Cu disks were subjected to HPT from 0.5 to 250 turns under a pressure of 6.0 GPa at room temperature. The V layers became thinner and fragmented with increasing numbers of HPT turns but finally mixed well with the Cu matrix throughout the disk samples. After 200 turns HPT processing, the nanostructured Cu-V alloy displays a submicron level heterostructure with a mixture of coarser grains (~ 100 nm and high Cu content) and finer grain (~ 20–30 nm and high V content). An ultimate tensile strength (UTS) of 1300 MPa with 3.5% elongation was achieved in a sample subjected to 200 turns HPT processing and post-HPT annealing at 773 K for 1 h. Thus, the HPT-processed immiscible Cu-V alloy achieved not only a significant microstructural refinement but also a remarkable strength enhancement through the solid mixing of Cu and V at room temperature.

## Introduction

Ultrafine-grained (UFG) materials are now attracting significant attention because their exceptional properties cannot be achieved using conventional coarse-grained material (Valiev et al. 2000; Huang and Langdon 2013). The application of severe plastic deformation (SPD) during the processing operation is the most important procedure for fabricating UFG materials (Valiev et al. 2006; Valiev 2014) where SPD techniques are preferred over conventional deformation processes since there is no significant change in the overall dimensions of the samples (Edalati et al. 2022). This means in practice that SPD processing can increase the strain imposed on the sample without any limitation. Different SPD techniques such as high-pressure torsion (HPT) (Zhilyaev and Langdon 2008; Čížek et al. 2011; Huang et al. 2016a), equal channel angular pressing (ECAP) (Valiev and Langdon 2006; Iwahashi et al. 1996; Hashemi et al. 2023), twist extrusion (Beygelzimer et al. 2009, 2011) and multi-directional forging (MDF) (Nakao and Miura 2011; Ghosh et al. 2016, 2021; Ghosh and Mula 2020; Dasharath et al. 2017; Singh et al. 2016) have been extensively studied in a range of investigations. Additionally, some new modified SPD methods have been proposed based on these early studies such as tubular channel angular pressing (TCAP) (Faraji et al. 2011), planar twist extrusion (PTE) (Beygelzimer et al. 2011), expansion equal channel extrusion (Exp-ECAE) (Sepahi-Boroujeni and Fereshteh-Saniee 2015; Öğüt et al. 2021), thin-walled open channel angular pressing (TWO-CAP) (Şahbaz et al. 2020; Kaya et al. 2023) and twisted variable channel angular pressing (TV-CAP) (Özbeyaz et al. 2022) and Vo-CAP (Beytüt et al. 2025) in order to make them applicable for different sample types or to eliminate some of the limitations of the basic SPD techniques.

When the published data for SPD is examined, there is a clear superiority in the ECAP and HPT studies in terms of the overall performance compared with other SPD techniques. Furthermore, HPT has an advantage over ECAP due to its ability to apply more severe shear strain on the processed materials and therefore to produce both smaller average grain sizes (Zhilyaev et al. 2022) and a higher fraction of grain boundaries having high angles of misorientation (Wongsa-Ngam et al. 2013). A disadvantage of HPT processing is the smaller sample size and microstructural non-uniformity along the disk sample diameter. However, due to the special capacity of applying severe straining to achieve ultra-high shear strains, it is concluded that HPT is a powerful tool for new materials development using unique processing to exceptionally high strain levels that cannot be achieved by other SPD methods.

Over the last decade, a new trend has emerged in HPT studies in which different metals are bonded together. In one of the earlier studies, a half disk of pure copper (Cu) and a half disk of pure aluminium (Al) were subjected to HPT processing at 6.0 GPa and room temperature (Oh-Ishi et al. 2013). The results showed that alternative Al-Cu layered structures with well-defined Al/Cu interfaces were formed by a stacking sequence along the disk normal at an early stage of the process. Later, this approach was developed and used for the processing of different full disks using a sandwich-type configuration with the disks stacked above each other in the HPT facility. In the first report of this type, two pure Al and one pure Mg disk were stacked on top of each other as Al/Mg/Al and subjected to HPT processing (Ahn et al. 2015) and the results showed that increasing the numbers of HPT turns led to a more uniform Al–Mg mixture and small average grain sizes of ~ 190 and ~ 90 nm at the disk edge after 5 and 10 turns, respectively. This study was followed by others showing that increasing the numbers of HPT turns led to a more homogenous mixture and further refined the grain size (Nikulin et al. 2016; Ibrahim et al. 2017; Han et al. 2017, 2019, 2020, 2022; Kawasaki et al. 2018; Bazarnik et al. 2020; Hernández-Escobar et al. 2022; Wang et al. 2024; Wu et al. 2025).

The synthesis of hybrid materials by SPD is now recognized as an emerging direction for SPD processing (Rogachev et al. 2020; Beygelzimer et al. 2025; Kulagin et al. 2019). The instabilities in the interfaces between dissimilar metals introduced by HPT leads to microstructural refinement, a redistribution of phases and ultimately to mixing (Kulagin et al. 2017, 2018; Pouryazdan et al. 2017). An investigation was reported where a single stack, composed of thin Cu and Ta foils, was subjected to HPT processing for up to 150 turns (Ibrahim et al. 2017) and the results showed that the Ta and Cu layers started to break after 30 turns so that a sufficient intermix was not achieved even after 50 turns. Images by scanning electron microscopy (SEM) demonstrated that a homogenous distribution was accomplished after 150 turns. Hardness measurements showed that the HPT-processed Cu-Ta alloy has higher hardness values than pure Cu and pure Ta processed by HPT. Although an increasing annealing temperature reduced the hardness of the HPT-processed Cu-Ta, it nevertheless remained above the HPT-processed Ta. This suggests that the HPT-processed Cu-Ta has a high thermal stability which is attributed to the presence of a Cu-Ta solid solution and a fine dispersion of Ta nano-particles within the Cu-rich grains.

It is feasible to study the HPT processing of Cu-Ta using Cu/Ta/Cu stacked disks (Mousavi et al. 2020) since this is a more practical and easy operation. A homogenous distribution was accomplished after 150 turns and additionally the average crystallite size, hardness and ultimate tensile strength were reported as ~ 35–45 nm, ~ 350 Hv and ~ 1300 MPa, respectively. A study of the effect of HPT on a Cu/Mo/Cu stack led to the development of a mathematical model to describe the phenomena during HPT (Tavakkoli et al. 2021). According to the results, the stability of the laminates under HPT was determined by geometrically necessary dislocations (GND). Applying HPT to a Cu/Mo/Cu laminate up to 32 turns showed that the hardness increases in Cu-based and Mo-based solid solutions at the centre and edge with respect to increasing strain (Mazilkin et al. 2024). From these results, it was concluded that finely dispersed small Mo particles contributed to the increase in the hardness of the Cu phase by acting as an obstacle to dislocation glide. Due to this effect, the percentage hardness increase in the copper phase was higher than in the Mo phase.

Currently, there is a high demand for developing Cu alloys with an added element insoluble with Cu in order to achieve high strength and no conductivity reduction (Pantsyrny et al. 2010) because if the alloy elements dissolve in, or react with, the matrix it may reduce the overall conductivity of the matrix. When alloying elements have low equilibrium solubility, they can theoretically be dispersed as fine particles or remain as separate phases without extensively dissolving into the Cu matrix, thereby preserving electrical conductivity (Pantsyrny et al. 2010). Vanadium possesses several advantageous properties including higher hardness, low density (6.1 g/cm^3^ compared to 10.2 g/cm^3^ for Mo and 16.4 g/cm^3^ for tantalum), and low neutron activation cross-section (Jain 2020; Naboychenko et al. 2009). Furthermore, the Cu–V equilibrium phase diagram shows that only a few percent of copper dissolves in the vanadium solid (Elliott 1965) and vanadium also has no intermediate intermetallic phases with Cu and it exhibits a minimum value of mutual solubility of 0.08 wt.% in copper at 20 °C (Pantsyrnyi et al. 2011). These characteristics make V an attractive choice for strengthening Cu while potentially maintaining good electrical properties (Pantsyrny et al. 2010), with potential applications in high-strength electrical conductors for high-field magnets, electrical contacts in demanding environments, and components in particle accelerators where both conductivity and weight reduction are critical. However, it should be noted that severe plastic deformation processing such as HPT can induce non-equilibrium solid solution formation, which may affect the electrical conductivity differently than the equilibrium microstructures. Therefore, V was chosen in the present experiments to enhance the copper matrix, with the understanding that the effect on electrical conductivity in HPT-processed Cu-V alloys requires systematic investigation in future work.

There is a report on Cu-V nanocomposites fabricated by mechanical alloying and vacuum hot-pressed sintering technology (Wang et al. 2019). Although stacked V/Cu/V disks processed by HPT to only five turns were reported to reveal initial mixing between V and Cu layers (Rogachev et al. 2020), but at present there is no report describing the HPT processing Cu/V/Cu stacked disks to extra-large numbers of turns to achieve a solid mixing of Cu and V in the synthesis of bulk Cu-V metallic composites. Accordingly, in the present study Cu-V composites were fabricated from stacked Cu/V/Cu disks set initially in a sandwich format and processed by HPT and thereafter the evolution of the microstructure and the mechanical properties of the HPT-processed Cu-V composites were investigated and comprehensively evaluated. This work is the first to demonstrate solid-state mixing and the formation of a unique nanostructure at room temperature in Cu-V nanocomposites using HPT, thus contributing new insight and expanding the application range for HPT-based alloy development.

## Experimental materials and procedures

Rods of oxygen-free Cu (99.95 wt.%) and V (99.8 wt.%) were supplied by Smith Metal Centres Ltd. and Goodfellow Cambridge Ltd., respectively. Both the Cu rod and the V rod were first annealed for 1 h at temperatures of 673 and 1173 K respectively. Both the annealed Cu rod and the annealed V rod were cut into disks with diameters of 10 mm and thicknesses of ~ 1.1 mm and this was followed by grinding to thicknesses of 0.8 mm. Then a V disk was placed between two Cu disks in a sandwich-like configuration and the stacked disks were processed by HPT at room temperature through total numbers of turns, *N*, of 0.5, 5, 10, 20, 50, 100, 200 and 250 turns. The HPT process was conducted by applying a pressure of 6.0 GPa at room temperature with a rotation speed of 1 rpm under quasi-constrained conditions in a way that allowed a small outflow of material around the periphery of the disk between the two anvils (Figueiredo et al. 2012). With a rotation speed of 1 rpm and applied pressure of 6.0 GPa, typical temperature rises ranging from 5 to 50 °C are recorded for various alloys and pure metals (Pereira et al. 2014; Edalati et al. 2018). It is estimated that the temperature rise during HPT processing of the Cu-V alloy is not more than 50°C.

Following HPT, each processed disk was cut in half along the diameter using electro-discharge machining (EDM) under water to avoid causing any change in microstructure. The cut samples were mounted and ground using SiC papers with mesh sizes of 800, 1200 and 2500, respectively, and then they were polished using diamond suspensions of 3 and 1 µm, respectively. The cross-section of each disk was examined using an optical microscope (OM) to analyse the mixing behaviour of the Cu-V-Cu layers. In addition, X-ray diffraction (XRD) was conducted using a Rigaku MiniFlex 600 machine which utilizes a Cu Kα monochromatic beam (wavelength λ = 0.154 nm) at 40 kV and a tube current of 15 mA. The XRD measurements were carried out between 2θ of 10° to 90° with a 0.05° step size at a scanning speed of 5° min^−1^. The XRD data was taken across the disk diameter and thickness in the sample cross-section. A Zeiss EVO LS15 scanning electron microscope (SEM) and Oxford Instruments Energy-Dispersive X-Ray Spectroscopy (EDS) were used for back scattered electron (BSE) imaging of the samples followed by elemental analysis at 20 keV and 1000 pA probe current for taking BSE images and 200 pA probe current for EDS mapping and line scanning at an 8.5 mm working distance. A transmission electron microscopy (TEM) study was conducted using a JEOL 2200FS EFTEM/STEM operating at 200 kV where this offered high resolution at high magnifications and permitted a detailed analysis of the sub-structures developed from the 200 turns HPT process. Sample preparation for TEM was carried out using a focused ion beam (FIB) technique, targeting specific regions of interest near the edge of the cross-section of the 200 turns sample. Additionally, compositional differences were examined through chemical mapping with energy dispersive X-ray spectroscopy (EDS) which was performed during scanning transmission electron microscopy (STEM).

Microhardness mapping of the sample cross-sections was achieved using an FM-300 microhardness tester to examine the effect of the number of HPT turns on the hardness distributions. The hardness values were measured on the cross-sections at intervals of 0.15 mm along the disk diameters and at intervals of 0.075 mm from the bottom to the top of the cross-section with a load of 150 gf and a dwell time of 15 s. Based on the measured hardness values, colour-coded maps were constructed to reflect the hardness distributions with respect to the distance from the centres of the disks.

Post-HPT annealing was applied to the 200 turns samples by annealing in a furnace having an inert atmosphere for one hour at 773, 973 and 1173 K, respectively. Subsequently, similar hardness measurements and colour-coded maps were constructed for the post-HPT annealed samples to reveal the relevant changes in the hardness distributions.

Specimens for tensile testing were cut from the 200 turns samples in both the HPT-processed and post-HPT annealed conditions. Based on a reference study (Loucif et al. 2012), two tensile test specimens were extracted from each disk where these specimens were arranged symmetrically with respect to the disk centre. The tensile specimens had gauge widths and gauge lengths of ~ 1.0 and ~ 1.1 mm, respectively. The tensile testing was conducted using a Zwick 30 kN Proline testing machine with an initial strain rate of 1.0 × 10^–3^ s^−1^ at room temperature. Because the tensile specimens from HPT are too small to easily use the extensometer, the broken parts of specimens were put together under an optical microscope to measure the elongations. At least two tensile specimens were cut and tested for each condition (HPT-processed and post-HPT annealing) to ensure the reproducibility of the results. The peak stress difference between repeated samples is smaller than 3%, demonstrating the reliability of the tensile tests.

## Experimental results

It has been established in HPT that the equivalent von Mises strain, ε, may be estimated using the relationship (Zhilyaev and Langdon 2008; Valiev et al. 1996; Wetscher et al. 2004)

1 $$\varepsilon =\frac{2N\pi r}{h\sqrt{3}}$$

where *r* and *h* are the radius and height (or thickness) of the disk, respectively. The equivalent von Mises strains along the disk radii (centre to edge) with different numbers of HPT turns show the extremely large strain levels applied to the HPT-processed samples with higher numbers of turns. The following sections present the detailed results for the microstructure and the mechanical properties in the HPT-processed Cu-V samples.

### Microstructures of HPT-processed Cu-V

**Fig. 1:**
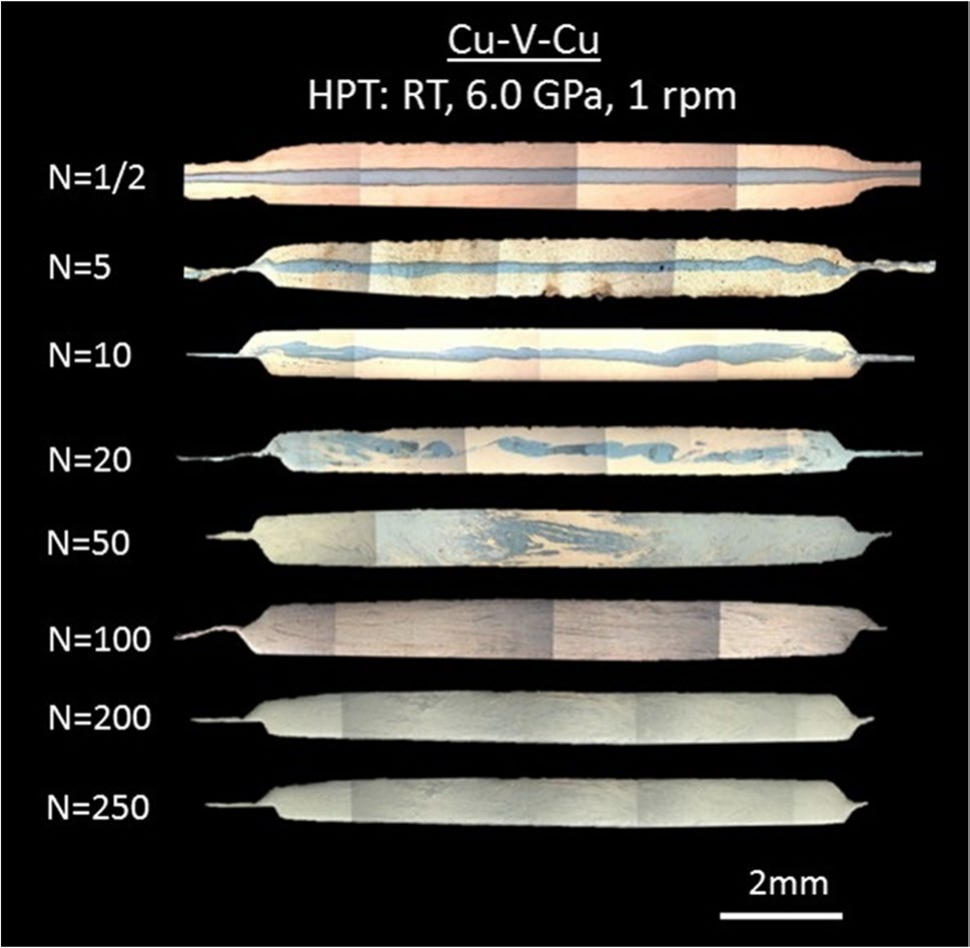
Cross-sections of Cu-V samples after HPT processing

Figure 1 shows OM images taken from the HPT-processed Cu/V/Cu stacks. The dark areas predominantly in the centres of the images are V-rich regions while the bright areas are Cu-rich regions. As can be seen in the images, the Cu and V layers are well-defined after 10 HPT turns and there is no evidence for any fragmentation of the V layer. After 20 turns of HPT there is fragmentation of the V layer in the half-radius to edge area and a general mixing between Cu and V in the disk edge area, whereas in the disk centre area there remains a clear distinction between the Cu and V layers. After 50 HPT turns there is a good mix between the Cu and V layers from the half-radius to edge area and this is readily apparent from the grey colour in the region. However, in the central region the thin and fine Cu-V interfaces remain visible even after 50 turns. After 100 turns there is generally a more homogenous mixture both in the disk edges and in the centre although some thin layers remain visible. With further increases to 200 and 250 turns there is essentially a full mix of Cu and V as is evident by the overall grey appearance of the cross-sections.

**Fig. 2:**
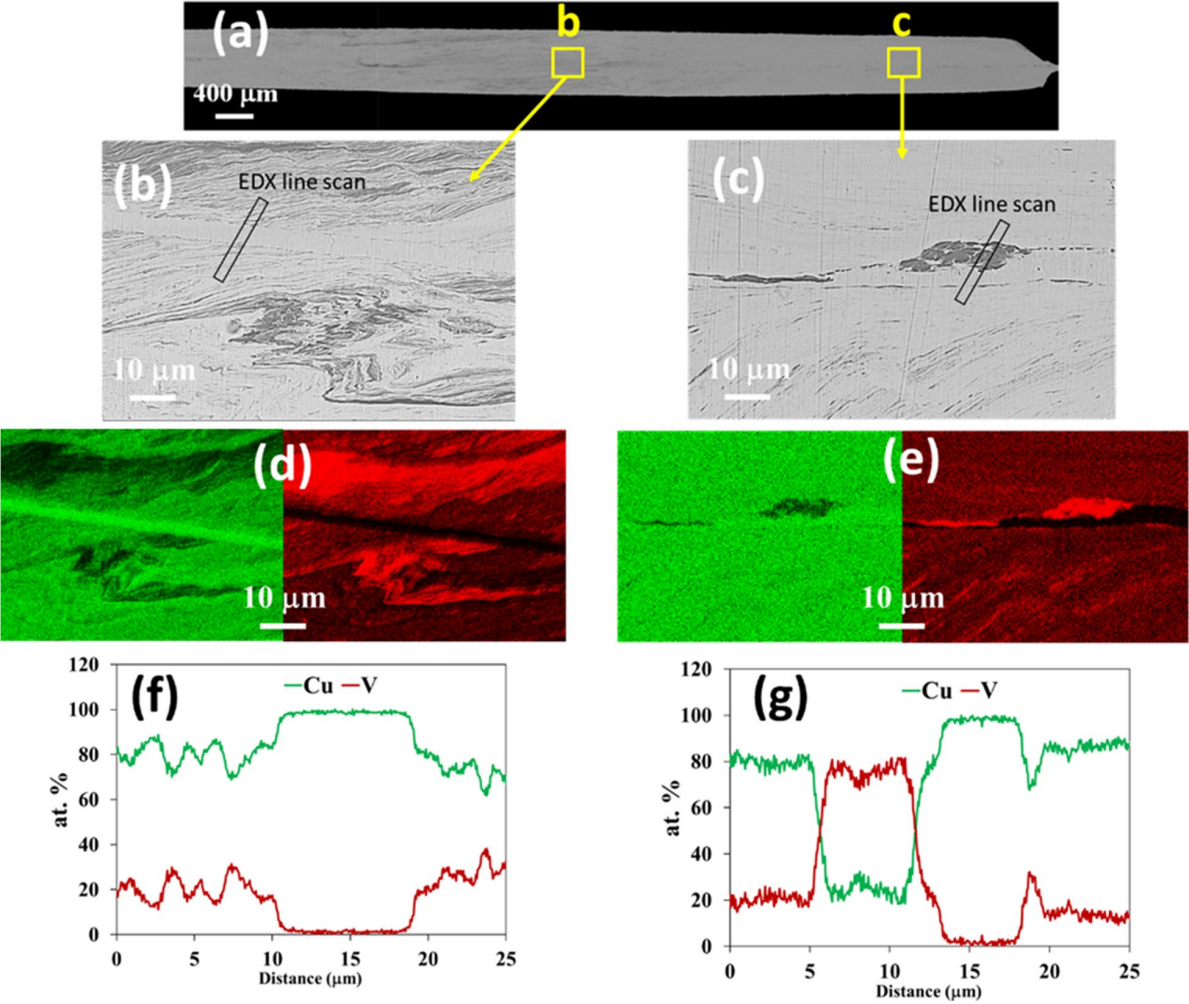
(**a**) Low magnification SEM image of the 200 turns sample, (**b**) and (**c**) high magnification SEM images of the selected areas, (**d**) and (**e**) elemental maps of the selected areas, (**f**) and (**g**) the EDX line scan data of the selected line

Figure 2 shows SEM images at different magnifications of the Cu-V sample processed by HPT through 200 turns. A low magnification SEM image of the cross-section of the 200 turns sample is shown in Fig. 2(a) and at this magnification the layer structures are less visible at both ends compared to the centre area of the sample. To evaluate the microstructure in more detail, high magnification SEM images from the area close to the centre and edge areas are presented in Fig. 2(b) and (c) and these images confirm the more homogenous nature of the microstructures at the edge areas. Thus, the central area shows a more curved layered structure whereas the edge areas show a slightly finer microstructure including a uniform matrix and fewer layers. This may be due to the higher strain applied to the edge area compared to the centre area of the disk sample during the HPT process.

Elemental mapping of Cu and V in the regions indicated by **b** and **c** are shown in Fig. 2(d-e). Thus, the microstructure corresponding to region b contains layers with high Cu content and some pure Cu layers are visible in this region, whereas at region c the microstructure includes Cu-rich and V-rich layers with no evidence for any layers of pure Cu. This shows that after 200 turns the Cu and V are mutually dissolved creating layers of solid solutions within the edge area. From the EDX line scans presented in Fig. 2(f), the V content in the Cu layers is between ~ 15–35% in the central region but at the edges the V content in the Cu layers is ~ 18–22% and the Cu content in the V layers is ~ 18–20% as shown in Fig. 2(g). This demonstrates that, contrary to general observations, the microstructure is not fully homogenous in this condition and further HPT turns are required in order to achieve a more homogenous microstructure across the sample.

**Fig. 3:**
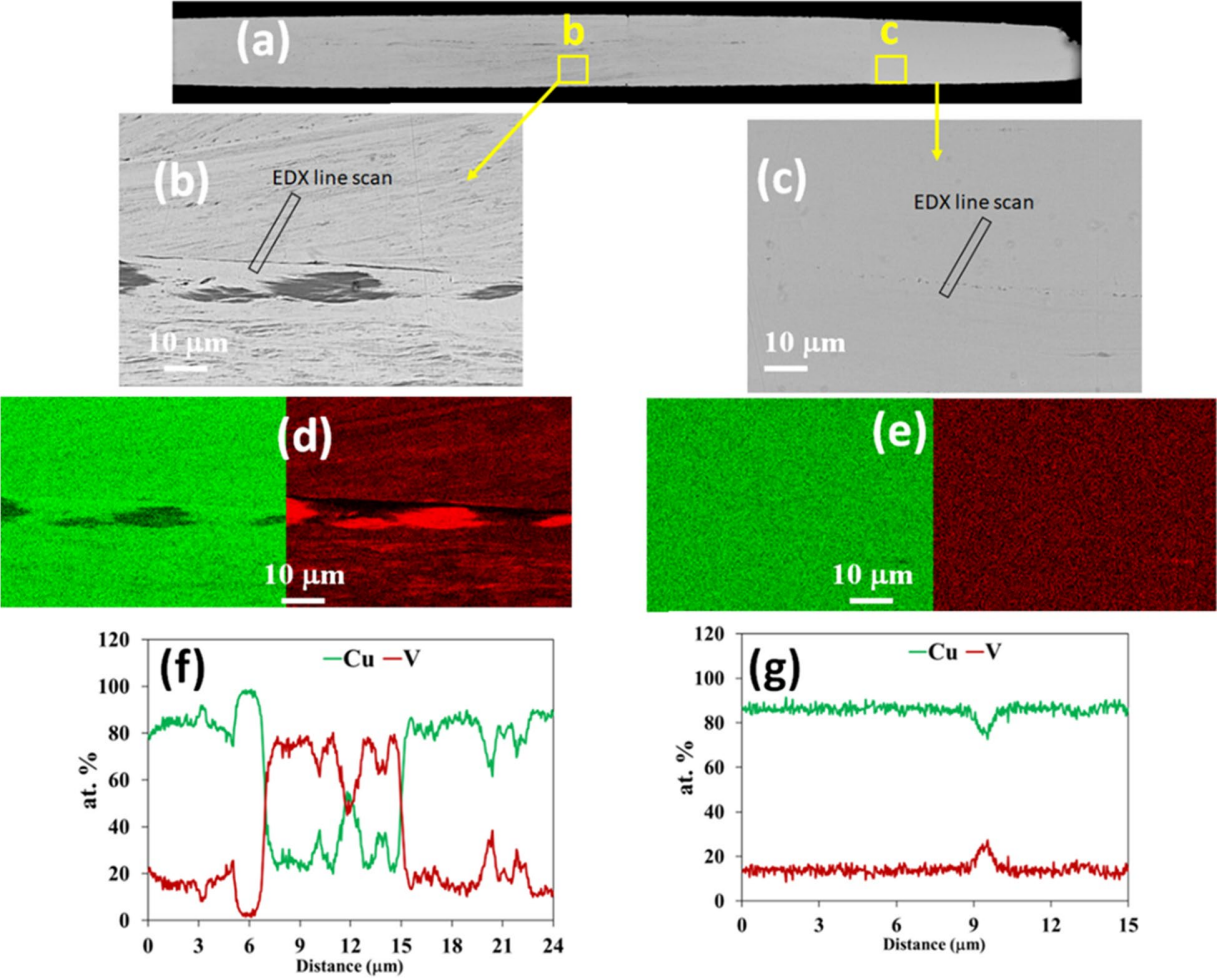
(**a**) Low magnification SEM image of the 250 turns sample, (**b**) and (**c**) high magnification SEM images of the selected areas, (**d**) and (**e**) elemental maps of the selected areas, (**f**) and (**g**) the EDX line scan data of the selected line

The microstructure after 250 turns is presented in Fig. 3(a-g). The low magnification cross-sectional SEM image shows more uniformity across the sample compared to the 200 turns sample and the high magnification SEM images from both the central and edge areas (Fig. 3(b-c)) show that the microstructure includes a uniform matrix with fine V-rich layers visible in the centre area but almost invisible in the edge area. Thus, the Cu–V boundaries almost fully disappear in the edge area of this sample. The EDX line data in the disk centre area, as shown in Fig. 3(f), confirms a uniform composition of Cu_80_V_20_ in the matrix with fine layers containing more V and having a composition of Cu_70_V_30_. Furthermore, Fig. 3(g) demonstrates that the disk edge area shows no evidence of any pure Cu or V-rich areas thereby indicating that the V has fully dissolved into the Cu with the formation of a uniform Cu-20 at% V solid solution. These findings indicate that a homogeneous microstructure with a matrix composed of Cu_80_V_20_ and thin V-rich layers can be produced by HPT processing by straining through 250 HPT turns. After HPT processing to 250 turns the pure Cu and pure V cannot be detected in the microstructure so that the mixture is complete to form a Cu-V alloy.

Based on the observations from Figs. 1, 2, and 3, it is concluded that the homogeneity in the microstructure increases as the number of HPT turns increases and ultimately, after a sufficient level of straining which was measured as ~ 250 turns, there is a very good mixing of Cu and V. This homogenization starts from the edge of the disk and expands towards the centre of the disk as the numbers of HPT turns increases.

**Fig. 4:**
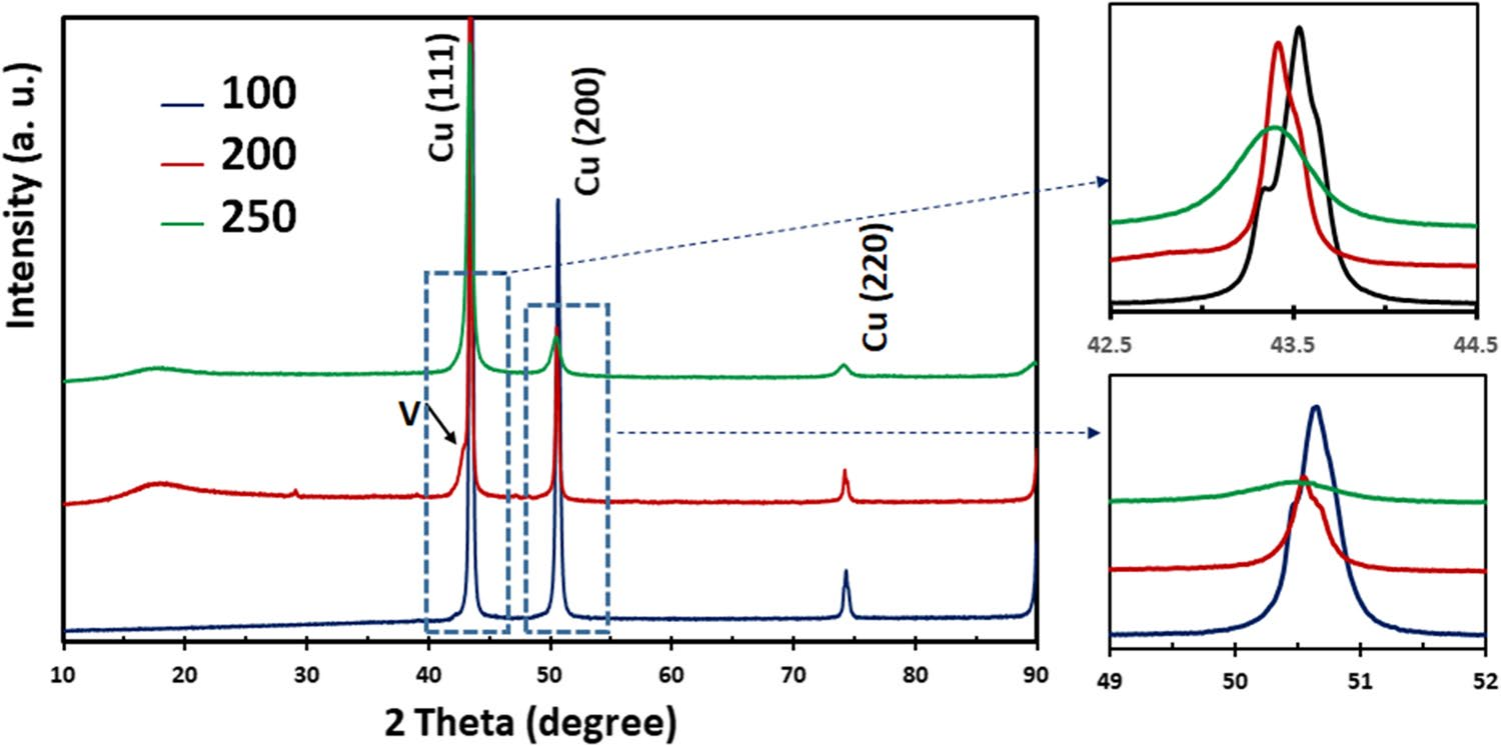
XRD patterns for the HPT processed Cu–V samples

The XRD patterns of the Cu-V samples processed by HPT through different numbers of turns (100, 200 and 250) are shown in Fig. 4. Inspection of these patterns shows that mainly the XRD peaks of Cu are visible. At 100 turns, the V peaks can be seen (for example, one small peak next to the Cu [111] peak) but they are weak with very small intensities. By increasing the numbers of HPT turns, the V peaks gradually disappear and only the Cu peaks remain. Additionally, an increase in the numbers of HPT turns causes a progressive decrease in peak intensities as well as a peak broadening, where this broadening is attributed to the refinement of the crystallite size and an increase in the lattice microstrain.

The Williamson–Hall equation (Suryanarayana and Norton 1998) was used to calculate the crystallite sizes and lattice microstrains for the Cu and the results are presented in Table 1. The crystallite size of Cu decreases by increasing the HPT turns and finally reaches a value of less than 20 nm after 250 turns whereas the lattice microstrain increases to ~ 1.7% after 250 turns. This level of refinement in the crystallite size to the lower levels of the nanoscale is attributed to the exceptionally high severe plastic deformation applied to the sample during processing.

**Table 1:** Crystallite size and microstrain of the Cu-V samples after 100, 200 and 250 turns HPT processing

	100 turns	200 turns	250 turns
Crystallite size (nm) (± 2)	90	34	12
Microstrain	0.9	1.4	1.7

After 100, 200 and 250 turns in Fig. 4, the XRD data show that the Cu diffraction peaks are shifted towards lower angles through increasing numbers of turns and the V peaks gradually disappear. This indicates that, with increasing HPT turns, more V dissolves in Cu to cause an increase in the lattice parameter of Cu. The atomic radius of the V atom (134 pm) is larger than the radius of the Cu atom (128 pm) and therefore the Cu crystal lattice grows as the Cu atoms are replaced by V atoms and this causes the peak to shift to lower angles. This provides additional confirmation that the HPT process can produce Cu-V alloys as seen in the SEM images in Fig. 3 where all EDX line data show the dissolution of V into Cu.

Materials Analysis Using Diffraction (MAUD) (Lutterotti et al. 1999) was used to calculate the average lattice parameter of the Cu–V system. An average V concentration of approximately 19.0% in Cu was calculated across the entire disk using Vegard's law (Denton and Ashcroft 1991) and it is noted that, consistent with the EDX data, this value is high when carrying out SPD processing that involves a bulk state reaction at room temperature. It should be noted that, according to the Cu-V phase diagram, Cu and V have a few percent solubility in each other (Elliott 1965; Pantsyrnyi et al. 2011). Therefore, the present investigation shows that the HPT technique provides a unique possibility of mixing these two elements at room temperature and thereby creating a new alloy with an unusual microstructure.

**Fig. 5:**
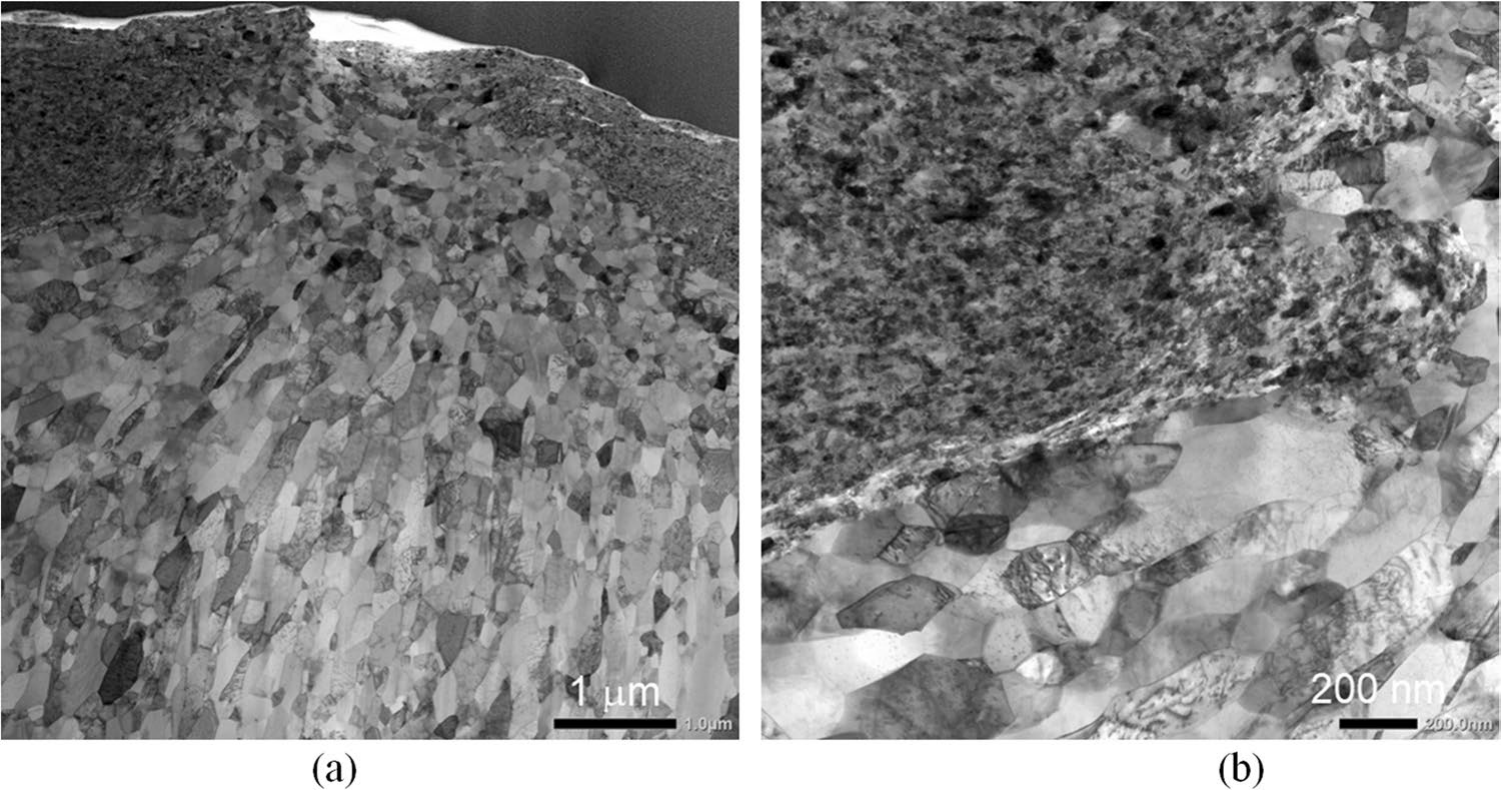
TEM images of the HPT-processed 200 turns Cu-V sample showing both (**a**) relative coarser grains area and (**b**) relative finer grain area

**Fig. 6:**
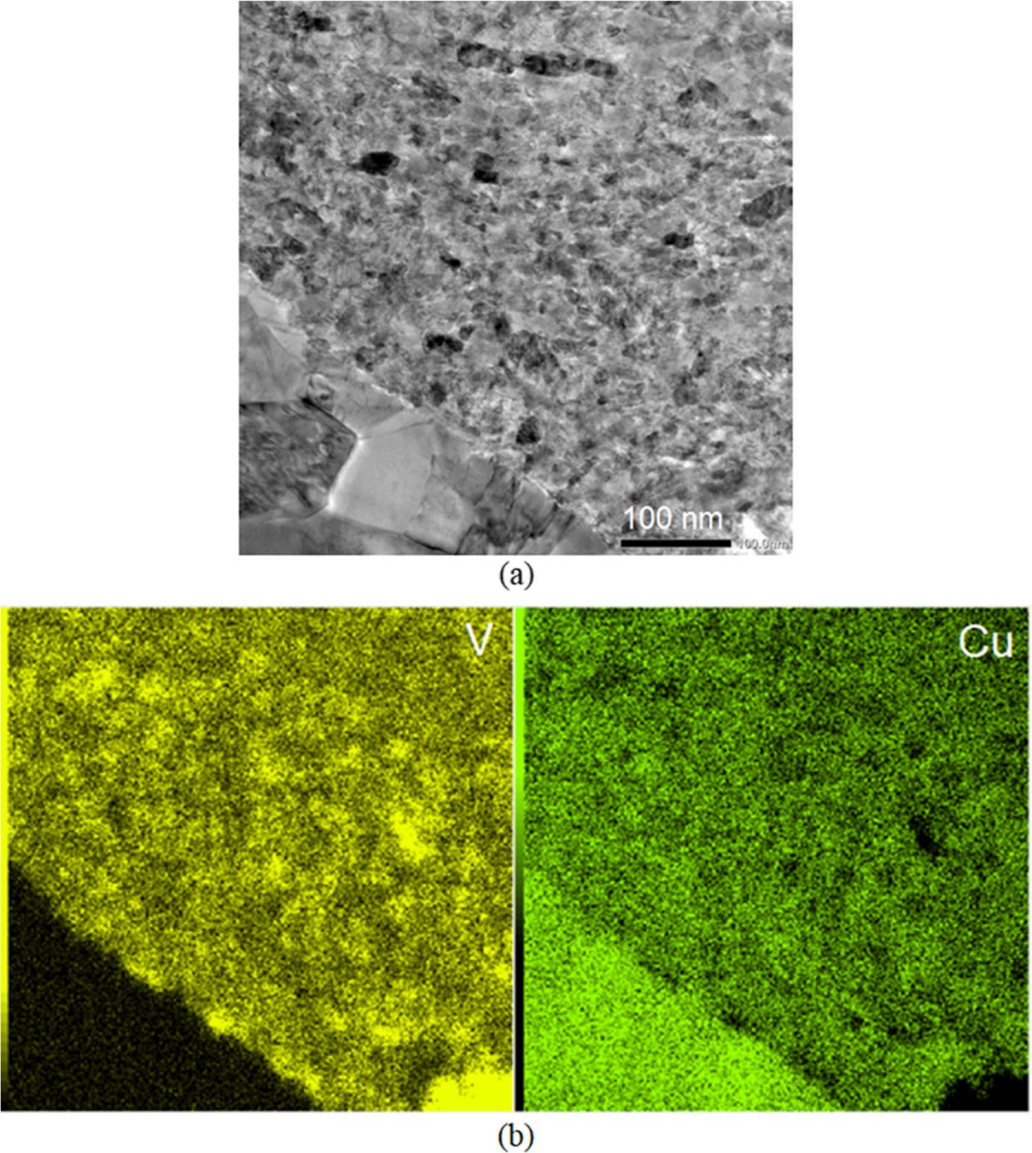
TEM images of the HPT-processed 200 turns Cu-V sample with (**a**) a region containing both relative coarser grains area and relative finer grain area, (**b**) the corresponding elemental mapping of V and Cu

**Fig. 7:**
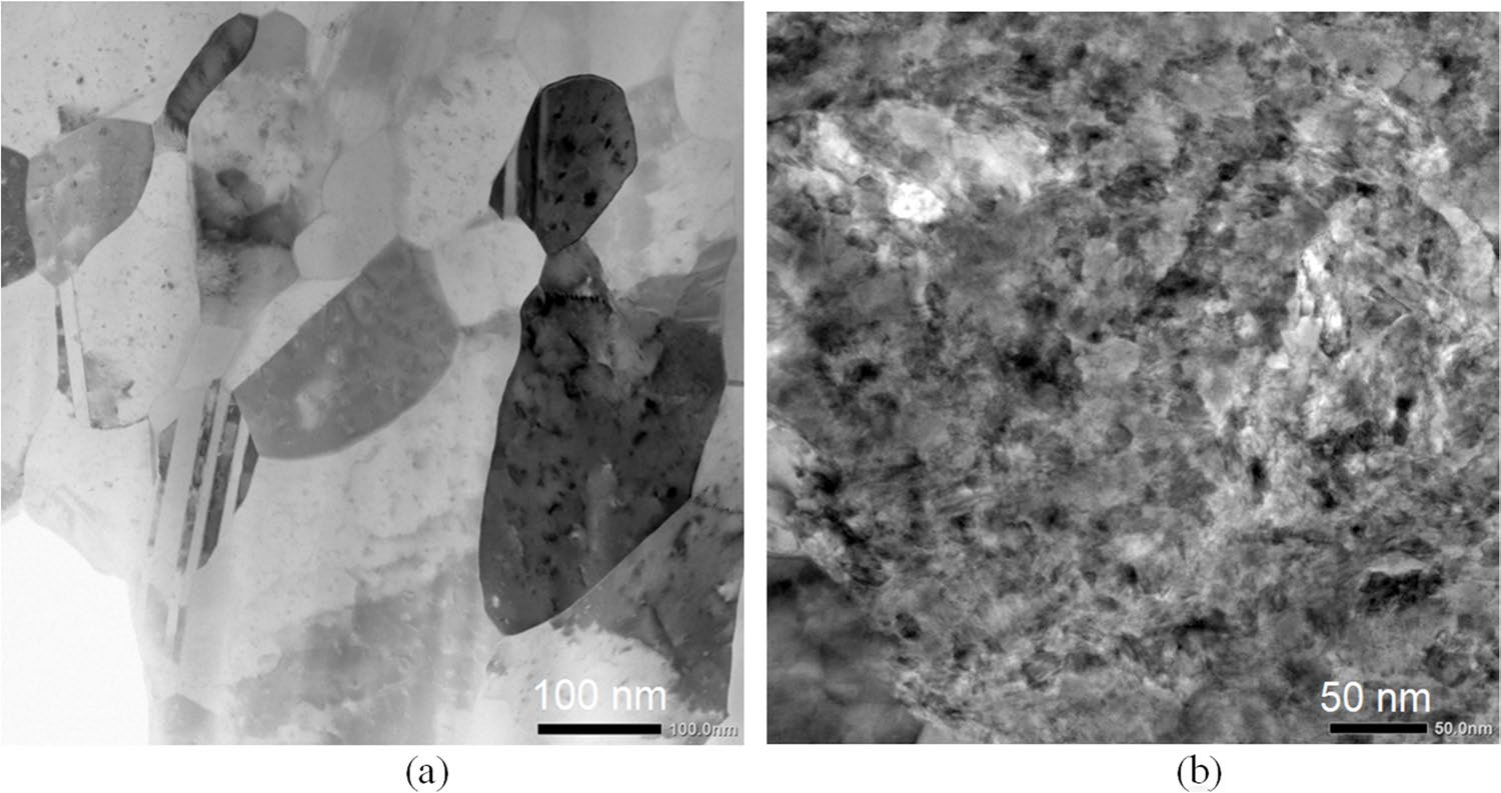
Higher magnification TEM images of the HPT-processed 200 turns Cu-V sample showing the microstructure details in (**a**) coarser grains area, (**b**) finer grains area

Microstructures of the HPT-processed 200 turns Cu-V alloy were further investigated using TEM observations. The low magnification TEM images in Fig. 5(a) and (b) show the heterogenous microstructure, some areas having relatively finer grains and some areas having relatively coarser grains. By increasing the TEM magnification and carrying out corresponding area element mapping on the elements V and Cu (yellow in V mapping and green in Cu mapping), as shown in Fig. 6, the relatively coarser grains have higher Cu and trace amount V content but the relatively finer grains have both Cu and V elements demonstrating a good mixing between Cu and V in this area. Figure 7(a) shows that in the relatively coarser grains (higher Cu and trace amount V content) at a higher magnification the grain size is ~ 100 nm and the presence of straight twins and fewer dislocations within these coarser grains indicates that dynamic recrystallization occurred during the HPT processing. The ultrafine grain size of ~ 100 nm in the relatively coarser grain area (higher Cu and trace amount V content) is consistent with the crystallite size of ~ 34 nm in the 200 turns sample which is listed in Table 1. By contrast, in the relatively finer grain areas with well-mixed Cu and V elements, as shown in Fig. 7(b), the grain boundaries are not well-defined and the grain size is estimated as ~ 20 − 30 nm. In summary, therefore, after 200 turns of HPT processing the fabricated Cu-V alloy has a Cu matrix which is made up of relatively coarser grains with higher Cu and trace amount V content and a grain size of ~ 100 nm and the relatively finer grain areas have well-mixed Cu and V elements with a grain size of ~ 20 − 30 nm. Similar grain size refinements have been reported for other systems (Zhilyaev and Langdon 2008; Zhilyaev et al. 2003) and provide further confirmation that HPT processing has a high potential for reducing the grain size to the lower nanometre range.

### Evaluation of mechanical properties

**Fig. 8:**
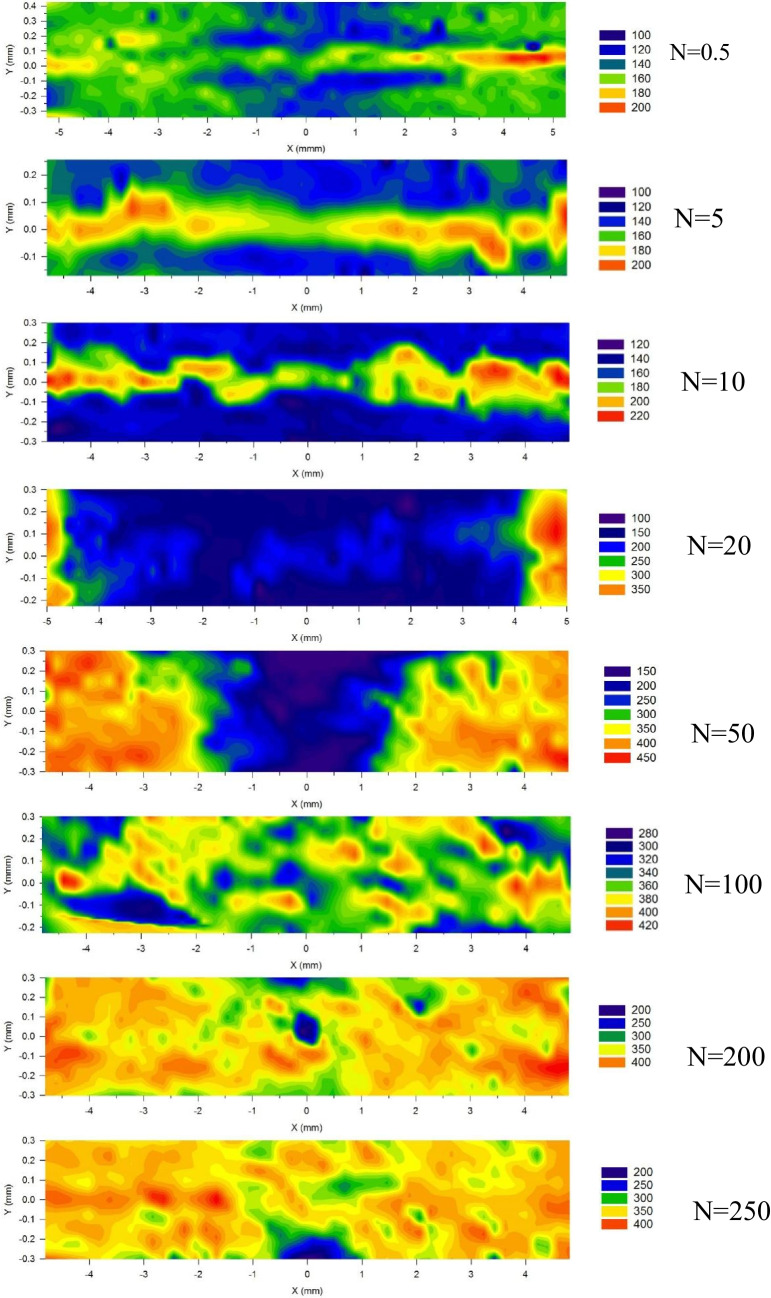
Hardness mapping on cross-section of HPT-processed Cu-V samples

Microhardness values were measured on the cross-sections of each sample and the results are presented in the form of colour-coded maps in Fig. 8 where X represents the distance from the disk centre and Y represents the distance from the half-thickness in the cross-section. For the 0.5 turn sample, the hardness was distributed between ~ 100 − 200 Hv and there is a narrow strip of higher hardness at Y = 0 mm along the X direction which is related to the unfragmented V disk. The narrow higher hardness strips at half thickness continue to exist after 5 and 10 turns although the hardness values are slightly increased. After 20 turns the narrow strip of higher hardness at half thickness shows a discontinuity which matches the observation of the V layer fragmentation in Fig. 1. In addition, the hardness at the disk edge area has higher values in the range of ~ 250–350 Hv but the disk centre has relatively lower hardness values of ~ 150–250 Hv. The 50 turns sample has higher hardness values of ~ 300–400 Hv at the disk edge area and the centre area has lower hardness values of ~ 200–250 Hv but these values are higher than in the 0.5, 10 and 20 turns samples. After 100 turns the hardness distribution is more homogenous both along the disk diameter and along the cross-sectional thickness with overall hardness values in the range of ~ 300–400 Hv. With further increases in the numbers of turns, the hardness values in the 200 and 250 turns samples are ~ 350–400 Hv. This is higher than in the 100 turns sample and the hardness homogeneity also increases significantly after 200 turns. Although the average hardness looks very similar for the 200 and 250 turns HPT-processed samples, a slight hardness reduction is visible at the edge of the disk which suggests the occurrence of limited recovery during the HPT processing.

**Fig. 9:**
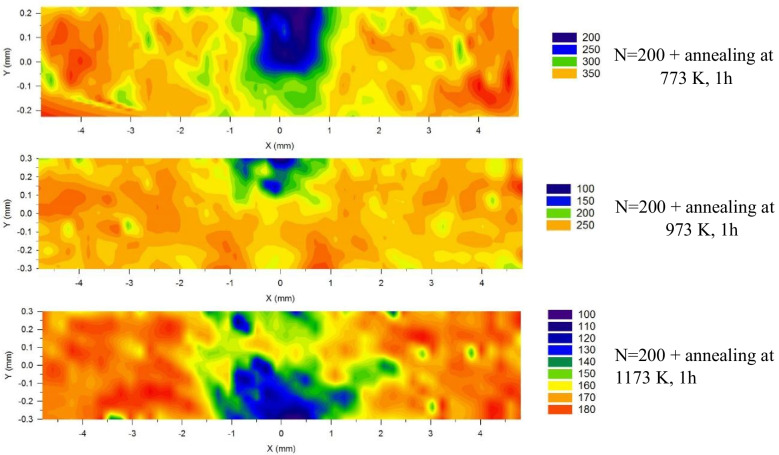
Hardness mapping on cross-section of 200 turns samples subjected to post-HPT annealing at 773, 973 and 1173 K

Figure 9 shows the hardness maps of the 200 turns samples subjected to post-HPT annealing. The hardness of the sample annealed at 773 K dropped from ~ 350–400 Hv to ~ 200–350 Hv and the region where the decrease in hardness was most intense was at the disk centre. A further increase in the annealing temperature led to a decrease in hardness which is apparent from the sample annealed at 973 K having a hardness ~ 150–250 Hv. A similar effect is visible on the sample annealed at 1173 K where the hardness is ~ 120–180 Hv. Thus, the hardness range in the 200 turns post-HPT annealed samples decreases with increasing annealing temperature.

**Fig. 10:**
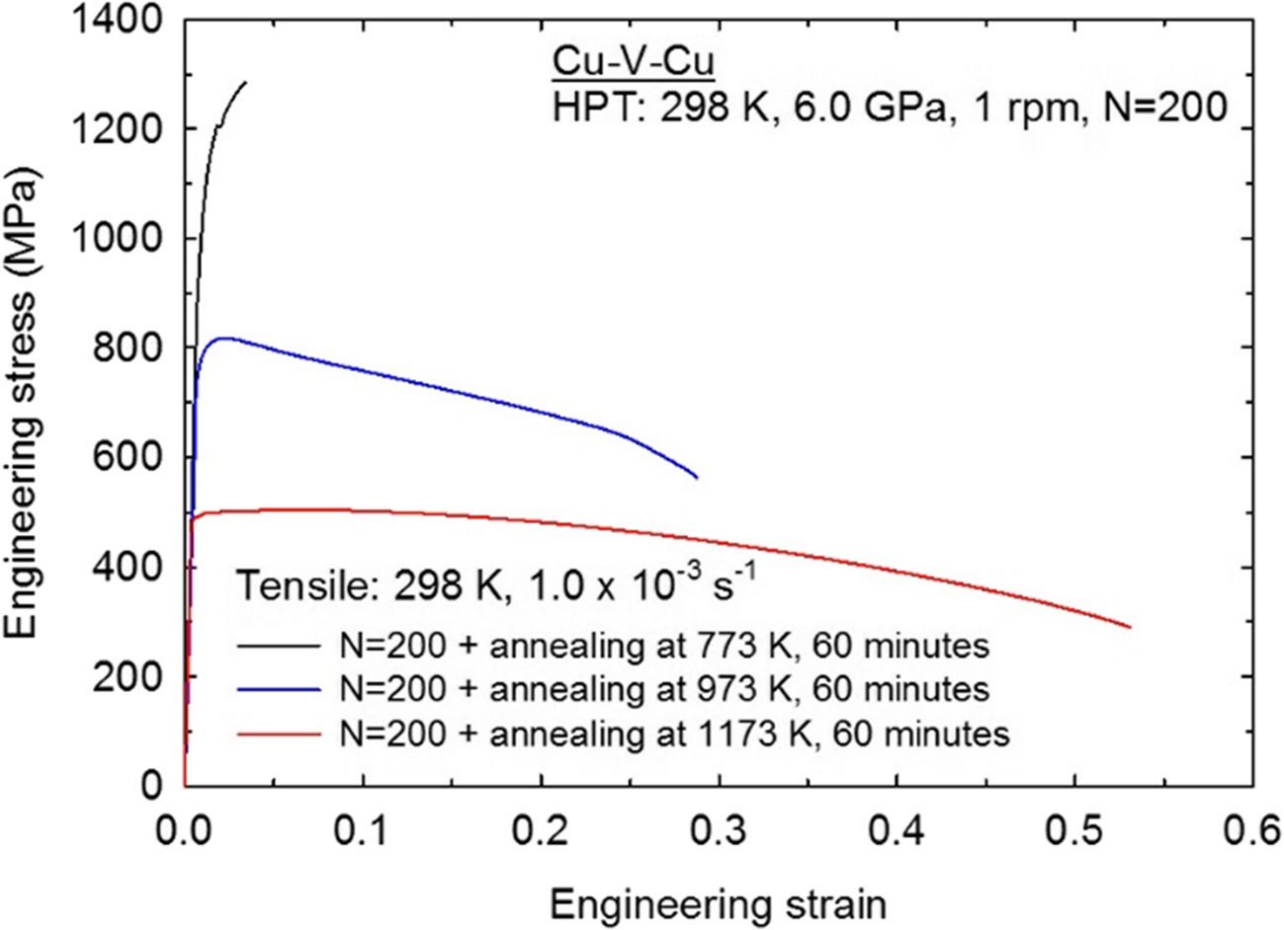
Stress–strain curves of 200 turns samples subjected to post-HPT annealing at 773, 973 and 1173 K

The engineering stress—engineering strain curves of the 200 turns samples are shown in Fig. 10 after post-HPT annealing at 773, 973 and 1173 K, respectively. The sample subjected to an annealing at 773 K displays a very high tensile strength of ~ 1300 MPa but a very limited ductility of only ~ 3.5% elongation. This high strength, together with the slight decrease in hardness only in disk central region, suggests that limited recovery or microstructural changes occurred during annealing at this low post-HPT annealing temperature of 773 K. With increased post-HPT annealing temperatures of 973 and 1173 K, the tensile strength drops to ~ 800 and ~ 500 MPa, respectively, and the ductility increases with elongations of ~ 30% and ~ 50%, respectively. This is related to the occurrence of static recrystallization and grain growth. A similar effect of post-HPT annealing temperatures on the mechanical properties is observed in the hardness results of the post-HPT annealed 200 turns sample as shown in Fig. 9.

## Discussion

### Microstructural evolution in the immiscible Cu-V system

It is now well established that the HPT technique enables the disk sample to undergo torsional straining under high pressure and this can lead to an ultrafine-grained microstructure. This high pressure plays a key role in successfully processing difficult-to-deform materials (Huang et al. 2012). In practice, significant grain refinement and the introduction of dislocations are the key elements in improving the physical and mechanical properties of HPT samples (Langdon 2013). The equivalent von Mises strain applied to HPT-processed samples can be estimated using Eq. (1), which indicates that HPT processing will produce inhomogeneities in both the microstructural and hardness distributions along the disk diameters. Nevertheless, it was reported that better microstructural and hardness distributions may be achieved with increasing numbers of HPT turns (Kawasaki et al. 2011) and this phenomenon was explained through the use of strain gradient plasticity modelling (Estrin et al. 2008). It follows that, in the present study, microstructural evolution occurs initially from the edges of the disks but then extends towards the centres as the numbers of HPT turns increases.

The present experimental results are based on using a Cu-V-Cu sandwich configuration and they show that the V layer in the Cu-V-Cu stack initially becomes thinner and then fragments into smaller pieces as the numbers of HPT turns increases. With increasing numbers of turns, the size of these small V-rich pieces decreases while a more homogenous distribution of these V-rich pieces occurs throughout the Cu matrix. To summarize, a microstructure with smaller V-rich regions is obtained at the edge of disk sample in the early stages of HPT but, with increasing numbers of HPT turns, a more homogeneous Cu-V alloy is obtained both along the radial direction and through the thickness of the disk. Thus, the findings of this study are consistent with other earlier studies (Zhilyaev et al. 2022; Han et al. 2020; Bazarnik et al. 2020) and they provide a clear demonstration that processing by HPT exhibits an important potential for fabricating copper-vanadium alloys.

The strain imposed on the Cu-V samples increases with increasing HPT turns and this leads to a refinement in the crystallite size and more crystalline defects so that additional diffusion paths are created for the Cu and V atoms. The XRD data in Table 1 shows there is a large reduction in the crystallite size of Cu, especially after processing through 200 HPT turns. This decrease in crystallite size continues with increasing numbers of turns such that a minimum crystallite size of ~ 12 nm was reached after 250 turns. This is a remarkably successful refinement compared to the results reported in earlier studies on other materials (Han et al. 2017; Bazarnik et al. 2020). Furthermore, this high level of reduction in the crystallite size produces an increase in the grain boundary fraction and hence leads to the formation of new diffusion pathways for the Cu and V which is analogous to the atomistic modelling proposed for Cu-Ta alloys (Koju et al. 2018). Additionally, it is apparent from the XRD data that the V-rich layers become finer with increasing numbers of HPT turns which thereby leads to a general mixing of the Cu and V.

The EDX data in Fig. 3 shows there is a uniform composition of Cu_80_V_20_ in the matrix. There is a report using a molecular dynamics simulation on an equilibrium immiscible Cu-V system (Shen et al. 2024) studying the structural phase transitions in the Cu-based solid solutions as a function of the V concentration. The simulation results revealed that the solid solution preserved an fcc crystalline structure until the V concentration exceeded a critical value of 23% (Shen et al. 2024) and this matches the present observations on the 20% V concentration in the Cu matrix after 250 turns of HPT processing. When these concentration values are compared with the results for the HPT-processed hybrid Cu-Ta system (Mousavi et al. 2020), it is readily concluded that these are exceptionally high solubility values for such a Cu-V immiscible system consisting of metals having different crystal structures. Furthermore, this observation becomes especially valuable when noting that this high solubility was achieved with a simple processing operation involving only a bulk-state reaction performed at room temperature.

Table 1 shows that the crystallite size decreases whereas the lattice microstrain increases with increasing HPT turns, with an average crystallite size of ~ 12 nm and a lattice microstrain of ~ 1.7 accomplished after 250 turns. These findings further support the earlier conclusion that processing through 200 turns is not sufficient to achieve a fully homogeneous microstructure.

### The microhardness and strength evolution

The hardness results in Fig. 8 show that the samples from 0.5 turn to 20 turns have a non-uniform hardness distribution where the maximum hardness is achieved in a narrow strip located between the Cu layers due to an unfragmented V layer. The hardness increases from the edges of the disks after 20 turns and those regions with higher hardness expand towards the centres of the disks as the numbers of HPT turns increase. These results are consistent with the OM images in Fig. 1 as the mixing between Cu and V starts from the edge and proceeds towards the centre. Figures 2 and 3 also reveal the difference in homogeneity in the microstructure, which supports the conclusion that a more homogeneous hardness distribution is obtained with increasing numbers of turns. Nevertheless, the average hardness also increases with increasing HPT turns. After 200 and 250 turns, very high hardness values ​​of ~ 350 − 400 Hv were achieved and these values are significantly higher than the value achieved for HPT-processed pure Cu (Chen et al. 2006) and also higher than HPT-processed pure V (Huang et al. 2016b).

The high hardness of the processed samples after 200 and 250 turns is attributed to the contribution of various strengthening mechanisms. First, there is solid solution strengthening in both the Cu-rich and V-rich areas. The Cu diffraction peaks shift towards lower angles in the XRD data as shown in Fig. 4, thereby demonstrating the lattice distortion produced by more V dissolving in Cu and the corresponding contribution to the material strength. Second, the interfaces between the Cu and the V layers provide additional barrier effects against dislocation movement and this leads to an increase in hardness. Also, the occurrence of grain refinement causes an increase in hardness in the Cu-rich and V-rich areas. The TEM observations on the 200 turns sample in Fig. 7 confirm that in the relatively coarser grain areas, having a higher Cu content, the grain size is around 100 nm whereas in the relatively finer grain areas with well-mixed Cu and V elements the grain size is estimated as around 20–30 nm. Finally, relatively finer grains with well-mixed Cu and V elements disperse within the Cu matrix and these may have a strengthening effect similar to the well-established precipitate hardening effect. Also, it is apparent that there is a limited recovery in the 250 turns Cu-V sample when considering the slight hardness reduction at the edges of the disk and this is consistent with an earlier study of vanadium (Huang et al. 2016b).

Post-HPT annealing applied to the 200 turns samples produced a reduction in the average hardness with increasing annealing temperatures. The lowest annealing temperature of 773 K gave a decrease in hardness only in the central region of the disk cross-section whereas with higher annealing temperatures of 973 and 1173 K the decrease in hardness gradually spreads to cover the entire cross-section. The hardness mapping in the HPT-processed samples in Fig. 8 shows that the 200 turns sample has slightly higher hardness values at the edge area than at the centre edge area. Furthermore, the cross-sectional observations in Fig. 1 also show fragmentation of the V layers and a general mixing of Cu and V starting from the edge area. This indicates that the mixing of Cu and V in the centre area is less good than in the edge area.

The thermal response of the HPT-processed Cu–V alloys is governed by the differential stability of their constituent phases and the spatial distribution of V. Earlier studies (Huang et al. 2016b; Jiang et al. 2000) showed that HPT-processed V has a better thermal stability at relatively low temperatures compared to the HPT-processed Cu. In the 200 turns sample, the heterogeneous V distribution creates distinct microstructural zones: Cu-rich regions with coarser grains (~ 100 nm) and Cu-V mixed regions with finer grains (~ 20–30 nm). This bimodal grain size distribution leads to differential thermal evolution during annealing. At 773 K, the Cu-rich regions, particularly in the disk centre where fewer Cu–V interfaces exist, undergo preferential recovery, resulting in localized hardness reduction. Conversely, the V-rich regions with finer grain size and higher solute content exhibit greater resistance to grain growth, maintaining their strengthening effect. For a further increase in the annealing temperature to 973 and 1173 K, both Cu and V phases undergo recovery and recrystallization, producing lower hardness values with a more homogenous hardness distribution across the disk. This heterogeneous microstructure offers potential advantages for property optimization. The coarser Cu-rich grains could provide improved ductility while the finer Cu-V mixed regions contribute to strength, achieving a favorable strength-ductility balance.

In contrast, the more homogeneous 250 turns sample, despite superior compositional uniformity, as shown in Fig. 8 exhibits slight hardness reduction at the disk edges compared to the 200 turns sample, suggesting that recovery was already initiated during the HPT processing thereby potentially limiting the thermal stability benefits of additional straining beyond 200 turns of HPT processing.

Based on the tensile results shown in Fig. 10, it is apparent that a very high UTS of ~ 1300 MPa was obtained in the 200 turns sample subjected to a post-HPT annealing at 773 K. This demonstrates that the UTS of HPT-processed Cu-V is higher than for HPT-processed pure V (~ 1200 MPa) (Huang et al. 2016b) or pure Cu (~ 400 MPa) (Alawadhi et al. 2017). Considering that the Cu-V alloys were simply fabricated from stacked Cu/V/Cu disks by HPT processing, the Cu is the matrix element of the HPT-processed Cu-V alloys and therefore it is reasonable to conclude that the smaller content of V makes a significant contribution to the high strength of the HPT-processed Cu-V alloy. As the post-HPT annealing temperature increases, the UTS value gradually decreases while the elongation at the point of failure increases.

Although electrical resistivity measurements were not conducted in this study, prior studies on Cu–V alloys produced by different synthesis methods suggest that the electrical conductivity remains largely retained because of the immiscibility of Cu and V and limited solubility of V in Cu. The maintenance of conductivity together with strength enhancement is thus anticipated in the present HPT-processed Cu–V alloys based on referenced literature (Pantsyrny et al. 2010; Pantsyrnyi et al. 2011; Wang et al. 2019). Future work will include direct resistivity measurements to quantitatively confirm these estimations.

## Summary and conclusions

1. A solid mixing of Cu and V was achieved through HPT processing at room temperature. The results demonstrate that it is possible to produce nanostructured immiscible Cu-V alloys by processing through 200 turns of HPT.
2. The processed microstructures in the 200 turns sample displayed heterostructured features at the submicron scale containing relatively coarser grain areas with a grain size of ~ 100 nm combined with relatively finer grain areas with a grain size ~ 20–30 nm.
3. Elemental mapping of the 200 turns sample at the submicron scale shows the local element distributions. Relatively coarser grain areas have higher Cu and trace amount V content whereas relatively finer grain areas have well-mixed Cu and V elements.
4. Hardness mapping on the HPT-processed samples show more homogenous hardness distributions as the numbers of HPT turns increased.
5. By applying post-HPT annealing at 773 K for 1 h on the 200 turns sample, a Cu-V alloy was produced with a UTS of ~ 1300 MPa which is higher than for HPT-processed pure Cu and pure V. Considering the stacked Cu/V/Cu disks processing and therefore overall high volume Cu and less volume V content in this immiscible Cu-V alloys, this result is promising for achieving strength enhancement in Cu based alloys.

## Data Availability

Data will be made available on request.
